# A Novel Mine Cage Safety Monitoring Algorithm Utilizing Visible Light

**DOI:** 10.3390/s20143920

**Published:** 2020-07-14

**Authors:** Xu Yang, Mingzhi Pang, Peihao Li, Pengpeng Chen, Qiang Niu

**Affiliations:** 1 School of Computer Science and Technology, China University of Mining and Technology, Xuzhou 221116, China; yang_xu@cumt.edu.cn (X.Y.); MingzPang@cumt.edu.cn (M.P.); ts18170012a31@cumt.edu.cn (P.L.); chenp@cumt.edu.cn (P.C.); 2 China Mine Digitization Engineering Research Center, Ministry of Education, Xuzhou 221116, China

**Keywords:** personnel counting, limb extension monitoring, visible light, safe area, mine cage

## Abstract

The mine cage has an important role in the production of coal mines. It has many safety problems in the transportation of people, such as overloading of personnel and illegal outreach of human limbs. However, the harsh mine environment makes it very difficult to monitor personnel overload and limb extension. To solve these two problems, we propose a novel safety monitoring algorithm of the mine cage based on visible light. With visible light technology, our algorithm cleverly utilizes the existing underground lighting equipment (i.e., miner’s headlamp and the miner’s lamp deployed on the mine cage) as the transmitter to broadcast the light beacons representing unique identity information through visible light frequency modulation. Next, cheap photodiodes deployed in the mine cage are used as the receiver to perceive the modulated optical signals. Then we use the frequency matching method for personnel counting and the frequency power comparison method for illegal limb extension monitoring. Moreover, a novel method of monitoring the delineated safe area of the mine cage is also proposed to ensure that all the miners are in the delineated safe area. Finally, we conducted extensive experiments with a simulated mine cage model. Results show that our algorithm has superior performance. With the photodiode SD5421-002, the accuracy of personnel overload judgment and safe area monitoring of our algorithm can reach 99%, and the accuracy of limb extension monitoring is more than 96%.

## 1. Introduction

The coal mine cage is a key transportation equipment in coal mine production for transporting miners, mineral resources, and mining equipment [1]. The miners have to be transported by the cages when entering the mine. Thus, the safety monitoring of the personnel in the cages is very important.

Although the function of the mine cage is similar to the elevator, the safety of the mine cage is very poor compared to an elevator due to the harsh mine environment. The problem of personnel load is one of the main factors affecting the safety of elevators, and this problem becomes more serious in the mine cage due to the harsh mine environment. The traditional method for monitoring elevator overload is to use gravity sensors [2]. Moreover, to prevent the failure of the gravity sensor, the method of using computer vision to count people in the elevator to determine whether the elevator is overloaded has attracted the attention of many researchers today [3,4,5]. However, the above-mentioned method for personnel overload monitoring including computer vision and gravity sensors is not applicable to coal mine cage environments. First of all, the underground mine is full of dust and water vapor [6], which means the visibility is extremely low in the underground [7], so it is difficult to use the computer vision method to count the number of personnel in the underground mine environment. Secondly, in addition to transporting personnel, mine cages also transport coal and other mineral resources, which makes the gravity sensor scheme invalid. Since the design of the mine cage is not completely closed, the door of the mine cage only prevents people or objects from falling through a few guardrails [8,9]. Thus, safety accidents often occur due to personnel limbs extend out of the cage. At present, the main methods used for limb perception are infrared sensors and some radio frequency technologies (e.g., Wifi, ZigBee, and RFID (Radio Frequency Identification)). First, since the special characteristics of high temperature, high humidity, and high dust concentration in the underground environment, the attenuation of traditional wireless signals is especially serious in the underground and hence directly leads to the ineffectiveness of underground personnel positioning algorithms that use traditional wireless signals. Second, radio waves (i.e., RFID, ZigBee, and WiFi) not only have serious multi-path effects, but also have expensive configuration costs, making its application in the underground personnel positioning more difficult.

To address this issue, we propose a novel safety monitoring algorithm for the mine cage utilizing visible light. This algorithm can effectively monitor the personnel overload of cages and illegal limb extension of miners. Our main idea is based on the emerging visible light technology. Compared with other wireless signals [10,11,12], visible light has the advantages of green safety [13,14], less multi-path effect [15], no electromagnetic effect [16,17,18] and strong resistance to electromagnetic interference [19,20,21]. The crucial thing is that the lighting equipment already present in the mine brings great convenience to our equipment deployment [22,23,24]. Nowadays, there are many innovative research papers and excellent results that apply visible light technologies, including visible light communications (VLC) technology and visible light positioning (VLP) technology to the underground mine environment, benefiting from the above-mentioned advantages of visible light. For example, the authors of [25] applied VLC to the underground mine for the first time, and proposed a Cell-ID positioning method through VLC technology. This method overcomes the problem that the traditional positioning system based on Cell-ID technique lacks accuracy due to the size and the density of cells by increasing the number of light sources. Similarly, in [26] and [27], two novel mine positioning methods were proposed by using VLC technology based on trilateration.

The main idea of our algorithm was to realize three goals, including personnel counting, limb extension monitoring, and safe area monitoring with the modulated LED lamps and cheap photodiodes (PDs). First, we utilized the miner’s headlamp as the transmitter and the PDs deployed on the top of the cage as the receiver, respectively. Then the frequency matching method was used for personnel counting. Second, the miner’s lamp deployed on the top of the cage door was used as the transmitter and the PDs deployed on the bottom of the cage door were used as the receiver, respectively. Then the frequency power comparison method was used for limb extension monitoring. Finally, we also propose a method of monitoring whether all the miners are in the delineated safe area of the mine cage for minimizing the probability of limbs extending out of the cage, which is achieved by the infrared LED lamp and PDs deployed in the designated cage safe area.

This paper makes the following three main contributions:

- We propose a novel mine cage safety monitoring algorithm. To the best of our knowledge, we are the first to propose the use of visible light technology to count miners with miner’s lamps. Besides, this method can also be applied to miner’s attendance in the future.
- We designed a special safe area monitoring method to minimize the probability of danger in the mine cage. To the best of our knowledge, we are the first to propose the use of visible light technology to perceive the range of human activity in underground mines. It can effectively reduce the probability of hidden risks only through the modulated LED lamp and a small number of PDs.
- We implemented our algorithm with a simulated coal mine cage. We also designed the special transmitter (including Xilinx ZYNQ-7020 FPGA and commercial LED lamps) and receiver (including the PD Honeywell SD5421-002 and AN706 digital-to-analog converter (ADC)). The experimental results show that the accuracy of personnel overload judgment and safe area monitoring of our algorithm can reach 99%, and the accuracy of limb extension monitoring is more than 96%.

The remainder of this paper is organized as follows. We first give an overview of our algorithm in Section 2. We then introduce the specific process of our algorithm in Section 3 and evaluates the effectiveness of our algorithm in Section 4. Finally, we conclude the paper in Section 5.

## 2. Algorithm Overview

**Figure 1:**
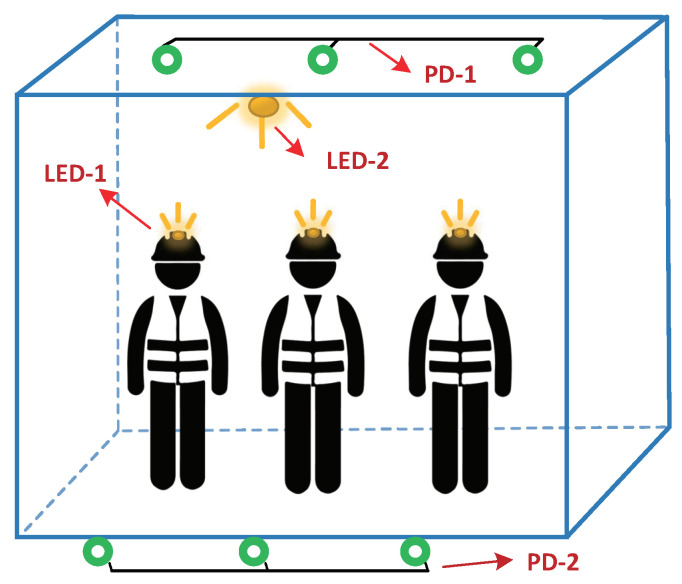
The usage scenario and the main idea of our algorithm.

Figure 1 illustrates the application scenario and the main idea of our algorithm. Our algorithm aims to provide effective safety monitoring for dangerous mine cage environment through visible light technology, especially in terms of personnel overload and illegal limb extension of miners. Thereby our algorithm is divided into two parts by default, i.e., the personnel counting part and the limb extension monitoring part. From Figure 1 we can see that the personnel counting part is implemented by the miner’s headlamps (i.e., LED-1) and a series of PDs deployed on the top of the cage (i.e., PD-1), and the limb extension monitoring part is realized by the miner’s lamp (i.e., LED-2) deployed on the top of the cage door and also a series of PDs (i.e., PD-2) deployed at the bottom of the cage door.

**Figure 2:**
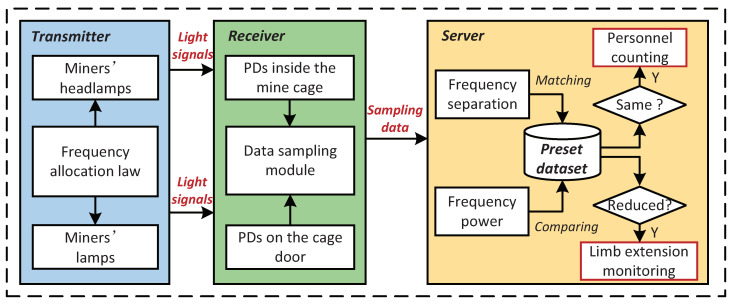
Algorithm workflow.

The basic workflow of our algorithm is shown in Figure 2. Similarly, we introduce the basic workflow of our algorithm separately based on the aforementioned two parts, i.e., the personnel counting part and the limb extension monitoring part. In particular, the workflow of the personnel counting part is as following steps:

- **Step 1:** We use pulse width modulation (PWM) to assign unique PWM frequencies to each miner’s headlamp (i.e., LED-1 in Figure 1) as their identity (ID) tags according to the frequency allocation law (Section 3.1.1) and then store the correspondence between miner *i* and frequency $f_{i}$ in the preset database.
- **Step 2:** When the miner enters the mine cage with his/her modulated headlamp on, the *j*-th PD deployed on the top of the cage (i.e., PD-1 in Figure 1) can perceive the modulated optical signals illuminating within its field of vision (FoV). Next, the data sampling module sends the sampled data of the voltage value of all the PDs to the server for processing.
- **Step 3:** After receiving the sampled data, the server separates all visible light frequencies of the mixed optical signals perceived by $P_{j}$, and the correct light frequencies can be matched by comparing with the preset dataset. Finally, we can accurately calculate the number of people in the mine cage combining all the PDs.

Then the workflow of limb extension monitoring is as following steps:

- **Step 1:** First, we modulate the miner’s lamp deployed on the top of the cage door (i.e., LED-2 in Figure 1) to broadcast the light beacon with unique PWM frequency, and we represent its flashing frequency as $f^{\prime }$.
- **Step 2:** Then we first collect fingerprints (i.e., the frequency power of $f^{\prime }$ perceived by the *k*-th PD when no limbs extend out of the mine cage) on a series of PDs deployed at the bottom of the cage door (i.e., PD-2 in Figure 1), and store these data into the preset dataset. Meanwhile we represent it as $P_{k}^{f\prime }$.
- **Step 3:** Finally, when the mine cage is running, the server calculates the real-time change ratio of the frequency power of $f^{\prime }$ obtained by each PD compared with the frequency power of the preset data (i.e., $P_{k}^{f\prime }$), and we regard the change ratio of the frequency power as the basis for limb extension monitoring.

## 3. Specific Process

In this section, we introduce the specific process of our algorithm. As before, we separately introduce the personnel counting part in Section 3.1 and the limb extension monitoring part in Section 3.2. At the same time, the safe area monitoring method is introduced in Section 3.3.

### 3.1. Personnel Counting

As mentioned earlier, we utilize the miner’s headlamps as the transmitter to broadcast each miner’s unique ID tag through visible light frequency modulation, thereby a series of visible light frequencies should be selected to be allocated to all miners. After the deployed PDs perceive the mixed optical signals with different visible light frequencies, the frequency separation method is used to obtain the ID tags of all the miners who reach the FoV of each PD. So we first introduce the frequency allocation method in detail and then introduce how to use the frequency separation method to count people.

#### 3.1.1. Frequency Allocation

We choose PWM to produce the visible light frequencies to distribute to all the miners. As a commonly-used visible light frequency modulation method in visible light communication (VLC), PWM can adjust the brightness of the LED light by controlling the proportion of the time the LED light is on [28,29,30], and we call this ratio the duty cycle [31]. Since the pulse waveform generated by the PWM is a square wave, from the previous work [32], the Fourier series expansion of the visible light pulse waveform with a duty cycle of *d* and a frequency of *f* is:

(1) $$f\left(t\right)=d+\sum_{n=1}^{\infty }\frac{2}{n\pi }\sin\left(\pi nd\right)\cos\left(2\pi nft\right)$$

It can be seen that the power emitted by the pulse wave is decomposed into the main frequency power (n=1) and countless harmonic components (n>1). In order to facilitate subsequent frequency separation and personnel counting, we introduce three constraints of frequency selection on this basis in turn.

**Duty cycle** As mentioned early, the duty cycle refers to the proportion of the LED lighting time in a blinking cycle. Assuming that the flashing frequency of an LED lamp is *f*, and the time that this LED lamp lighting is $T_{on}$, thereby the flashing period *T* of this LED lamp is 1/f, and the duty cycle *D* is $T_{on}\times f$. First of all, when the flashing frequency of the LED lamp is fast enough, the brightness perceived by the human eye is the average brightness value of the LED light during the power-on time and power-off time within a blinking cycle [33]. Therefore, the greater the duty cycle, the greater the brightness of the LED lamp will be [34], which can better meet the lighting requirements of miners. Second, we can infer from Equation (1) that the number of harmonics is related to the duty cycle *d*. Note that when d=50%, Equation (1) becomes:

(2) $$f\left(t\right)=\frac{1}{2}+\sum_{n=1}^{\infty }\frac{2}{n\pi }\sin\left(\frac{\pi n}{2}\right)\cos\left(2\pi nft\right)$$

when *n* is even, we have:

(3) $$\sin\left(\frac{\pi n}{2}\right)=0,f\left(t\right)=\frac{1}{2}$$

**Figure 3:**
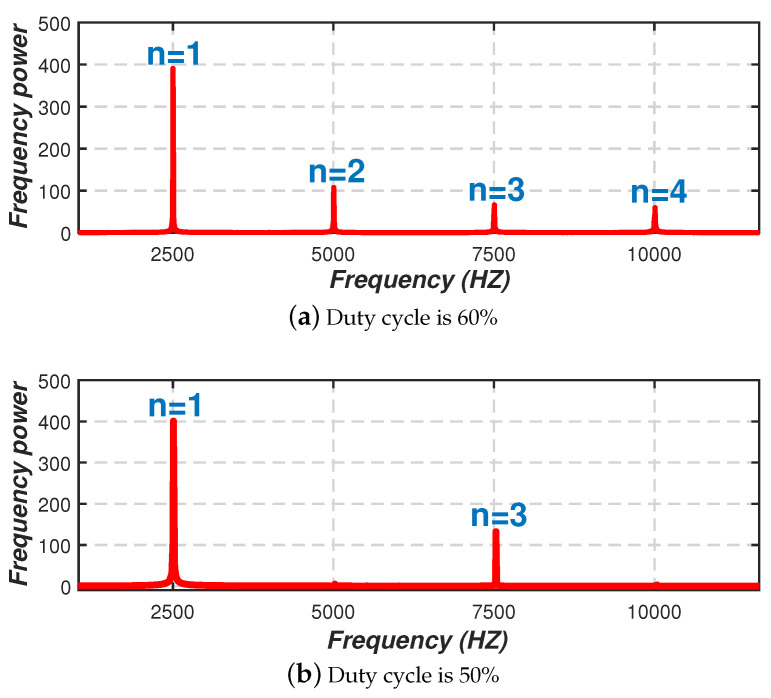
Relationship between duty cycle and number of harmonics.

It indicates that when the duty cycle d=50%, the number of even harmonics is 0, and only odd harmonics exist. Figure 3 shows the spectrum of a square wave signal with a PWM frequency of 2500 Hz under different duty cycles after fast Fourier transform (FFT), where the *x*-axis represents the visible light frequency and the *y*-axis is the frequency power. It can be seen that when the duty cycle is 60%, odd harmonics and even harmonics coexist. When the duty ratio is 50%, only odd harmonics exist in the corresponding spectrum diagram at this time, and the number of harmonics is half of that when the duty cycle is 60%. Thus, in order to weaken the impact of excessive harmonics on the subsequent frequency separation, and take into account the lighting effect at the same time, the best effect can be achieved by setting the duty cycle of the miner’s lamp flashing to 50%.

**Figure 4:**
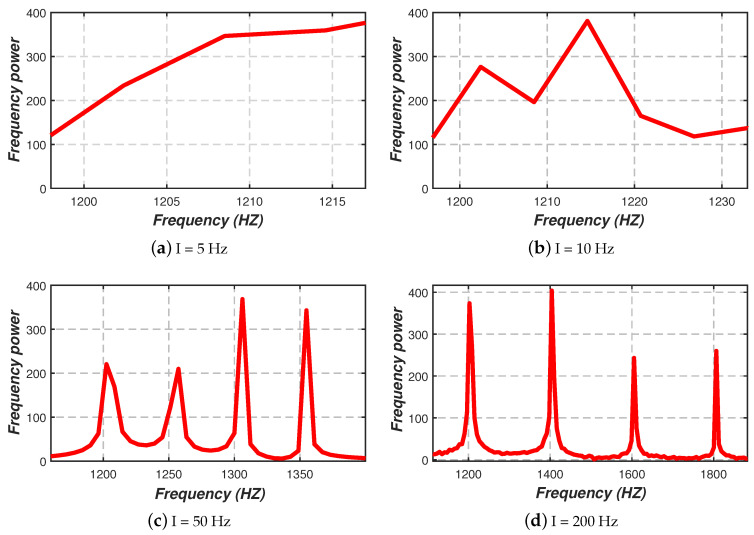
The effect of different adjacent frequency intervals on frequency recognizing, where I represents the adjacent frequency interval.

**Frequency threshold***First of all,* the human eye has the problem of frequency flicker [35]. According to the previous work, when the flicker frequency of the LED lamp is less than 1000 Hz, the human eye may perceive the flicker of LED lamps, thereby interfering with the human eye and further affecting the lighting effect [36,37]. *Secondly,* the Nyquist sampling theorem states that only when the sampling frequency is greater than twice the highest frequency in the signal can the original information in the signal be completely retained [38]. That is, assuming that the sampling frequency is *R*, then the visible light frequencies we set should be less than R/2. It means that the upper limit of the number of PWM frequencies we can choose is determined by the sampling frequency (i.e., *R*). *Thirdly,* due to the mutual influence between adjacent frequencies, the interval between the adjacent frequencies we set must be greater than a certain minimum threshold to ensure reliable frequency separation of the visible light [39]. Now, we choose the best adjacent frequency interval. Figure 4 shows the spectrum obtained by FFT after the mixed optical signals of four LED lamps at four different adjacent frequency intervals are collected by PD. We can observe that the power peaks of the four frequencies we set are not obvious when the adjacent frequency interval is 5 Hz, and the correct visible light frequencies we set is unrecognizable at this time. Next, when the adjacent frequency interval is 10 Hz, we still can not match these frequency power peaks with the set four frequencies correctly though there are more obvious frequency power peaks. Then it can be seen that when the adjacent frequency interval is 50 Hz, the power peaks corresponding to each visible light frequency are already clear, and when the adjacent frequency interval is 200 Hz, we can identify each frequency’s power peak accurately. Therefore, we set the adjacent frequency interval of the miner’s headlamps to be greater than 200 Hz to ensure sustainable and reliable visible light frequency separation. *Finally,* we conclude that the range of the visible light frequency we set is between 1000 Hz and R/2, where R is the sampling frequency. The adjacent frequency interval of the miner’s headlamps is greater than 200 Hz.

**Figure 5:**
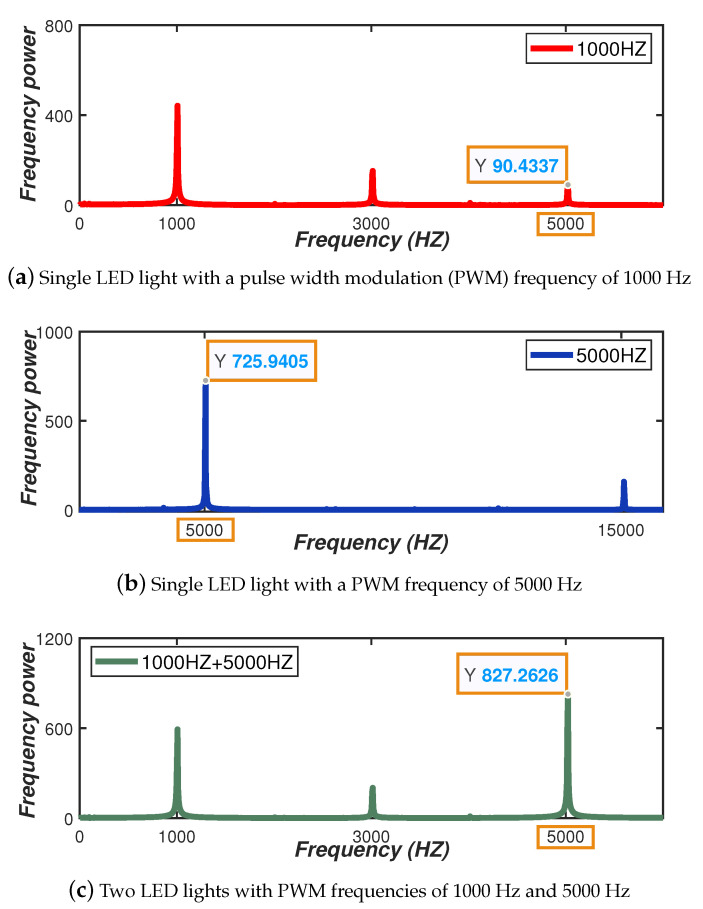
The effect of frequency multiple and corresponding frequency power.

**Frequency multiple** As mentioned earlier, the power emitted by the pulse wave generated by PWM modulation of a single LED light will be decomposed into the power of the main frequency and the power of countless harmonic components. Assuming that the frequencies of the two headlamps are $f_{1}$ and $f_{2}$, respectively, and $f_{1}$ is an integer multiple of $f_{2}$. Thereby the frequency power of $f_{1}$ is likely to overlap with the corresponding harmonic frequency power of $f_{2}$. We show in Figure 5 the spectrum obtained by using two LED lamps with PWM frequencies of 1000 Hz and 5000 Hz, respectively. When two LED lamps with PWM frequencies of 1000 Hz and 5000 Hz exist at the same time, the frequency power corresponding to 5000 Hz in the obtained spectrum is the approximate sum of the frequency power of 5000 Hz corresponding to the two single LED lamps (since there is the effect of frequency power fluctuations, it is approximately equal); therefore, the visible light frequencies we set cannot be multiple relationships.

#### 3.1.2. Frequency Separation and Personnel Counting

**Figure 6:**
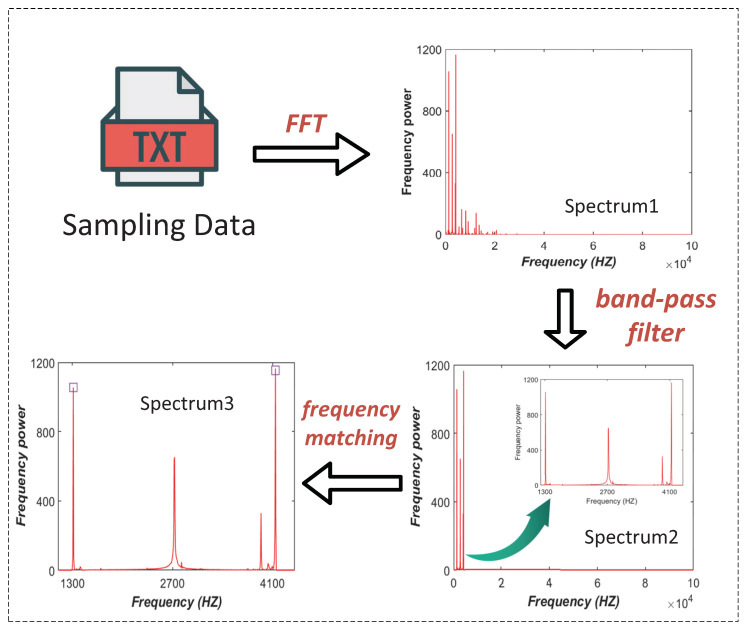
Frequency separation of two LEDs with the PWM frequencies of 2300 Hz and 2500 Hz.

An effective frequency separation method can ensure the accuracy of the personnel counting of our algorithm. Next, we introduce the specific steps of the frequency separation. Firstly, the system continuously samples and saves the voltage data of all PDs deployed on the top of the cage. Secondly, we separate all visible light frequencies and corresponding power perceived by each PD through FFT. Then the band-pass filter is used to filter out the harmonics and other clutter frequencies that are not in the preset frequency range. Finally, the correct miner frequencies received by each PD are obtained by matching with the preset dataset. Then by combining all PDs, efficient and accurate personnel counting in the mine cage can be achieved. Figure 6 depicts a simple frequency separation process of two set PWM frequencies, in which we selected two LED lamps with PWM frequencies of 1300 Hz and 4100 Hz as the miner’s headlamps carrying their ID information, respectively. Furthermore, one LED lamp with a modulated frequency of 2700 Hz was used as the external light interference. The spectrum obtained after FFT processing is shown as Spectrum 1 in Figure 6. It can be seen that the obtained visible light frequencies is very messy due to the presence of a large number of harmonics. Since the modulated frequency assigned to each miner is known, we use a band-pass filter to reserve the frequency range of 1300–4100 Hz. It can be seen from Spectrum 2 that the band-pass filter has filtered out many harmonics and other uncorrelated frequencies, leaving only the frequency range we need. On the other hand, there is also a case where the spectrum obtained after processing by the band-pass filter still has power peaks of other frequencies within the reserved frequency range, e.g., the clutter with a frequency of 2700 Hz and the harmonic with a frequency of 3900 Hz in Spectrum 2. To solve this problem, We only need to match the reserved frequencies with the preset dataset to determine the correct frequencies, as shown in Spectrum 3.

### 3.2. Limb Extension Monitoring

We utilize the miner’s lamp deployed on the top of the cage door and the PDs deployed on the bottom of the cage door to perform limb extension monitoring. The main idea is as follows. Firstly, the frequency allocation method introduced earlier is used to allocate to the miner’s lamp. In order to avoid the mutual interference between the miner’s headlights and the miner’s lamp, we set the rules for frequency selection of miner’s lamp as follows. In the case where the frequency of the miner’ headlamps and the miner’s lamp meet the frequency allocation method, we mark the set of headlamp frequencies for *n* miners as $f_{1},f_{2},f_{3},\cdots ,f_{n}$, and the frequency of the miner’ lamp as $f_{a}$. Then we only need to ensure that the frequency of the miner’s lamp is greater than the frequency of all miner’ headlamps (i.e., $f_{a}>max\left(f_{1},f_{2},f_{3},\cdots ,f_{n}\right)$), so that we can use the band pass filter to filter out the unrelated frequencies. Secondly, when the light irradiated by the miner’s lamp at the top of the cage door with a frequency of $f^{\prime }$ illuminating the PD is blocked by the object, the light intensity perceived by the PD will decrease. Thirdly, since the light intensity at the frequency $f^{\prime }$ sensed by the PD is proportional to the frequency power at $f^{\prime }$, so when the intensity of $f^{\prime }$ decreases, the corresponding frequency power will also decrease [40]. Finally, we can determine whether the light is blocked by external objects (i.e., whether the person’s limb is out of the mine cage) through the change of the frequency power of $f^{\prime }$. Note that we are monitoring the frequency power of a specific frequency (i.e., $f^{\prime }$), so the light in the external environment will not affect the monitoring effect of our algorithm. Next, we introduce the specific process of limb extension monitoring.

**Figure 7:**
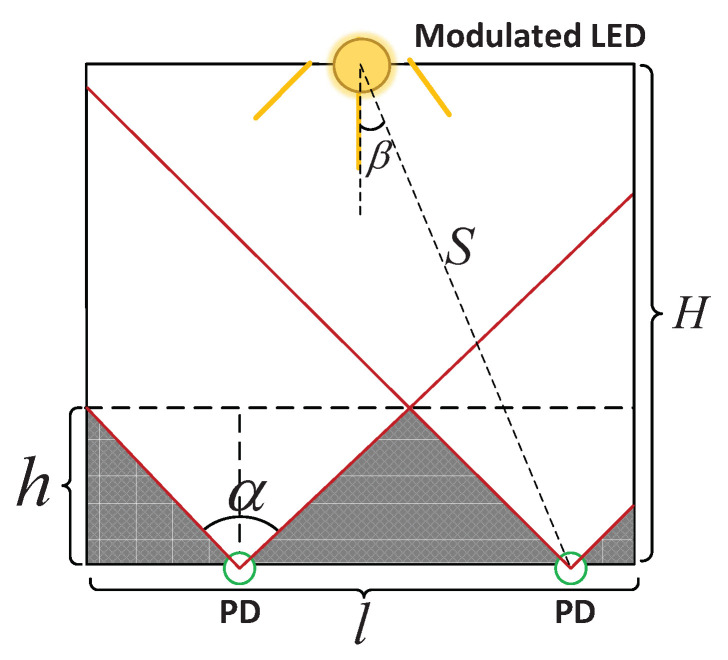
2D plan of the mine cage, where the shaded parts indicate the perception blind zone of photodiodes (PDs).

(1) We first collect the frequency power $P_{k}$ of the miner’s lamp on the cage door as perceived by the *k*-th PD deployed at the bottom of the cage door without the obstruction of external objects and then store it in the preset dataset.
(2) Assuming that when a person enters the cage, the frequency power of the *k*-th PD at time *t* is $P_{k}\left(t\right)$, then the change ratio of the frequency power of the *k*-th PD can be expressed as:
  (4) $$P_{k}^{change}\left(t\right)=\frac{P_{k}-P_{k}\left(t\right)}{P_{k}}\times 100\%$$
  Since the fluctuation of the light intensity, the inherent noise of the PD, and the incomplete waveform factors generated by the LED, the frequency power fluctuation rate is less than 20% when the light is not blocked [32]. In order to maximize the accuracy of the limb extension monitoring of our algorithm, we think that when any PD receives a frequency power change rate of $f^{\prime }$ is greater than 20%, it indicates that the limb has extended out of the cage.
(3) Due to the limitation of the sensing angle (i.e., FoV) of the PD, in theory, the more PDs are deployed, the higher the accuracy of the limb extension monitoring. On the other hand, it is not practical to deploy a large number of PDs in a harsh mine cage environment. Thus we need to give the best number of deployed PDs according to the actual situation of the mine cage. The conditions for the deployment of PDs are as follows.
  - **Condition 1:** As shown in Figure 7, in the case where the miner’s lamp can fill the bottom of the entire cage door (see **Step 3**), assume that the minimum height we need to monitor is *h*, the width is *l*, and the FoV of the PD is α, then the number *N* of PD we choose is at least:
    (5) $$N=\lceil \frac{l}{2h\tan\frac{\alpha }{2}}\rceil$$
  - **Condition 2:** The larger the FoV of the PD, the smaller the blind zone for sensing light, so the fewer PDs are needed. Half of the FoV of the PD should be greater than the angle β between the connection between the PD and the LED and the plane normal to the LED to ensure the PD’s effective reception of the modulated light signal. We express the height of the mine cage as *H*, and the distance from the deployed PD to the mine cage as *S*, therefore we give the selection range:
    (6) $$\frac{\alpha }{2}\geq \arccos\frac{H}{S}$$
  - **Condition 3:** Since most of the miner’s lamps are spotlights with a certain spotlight angle, we need to ensure that the illumination range of the LED lamp deployed on the top of the cage door can fully cover the PDs deployed at the bottom of the cage door. We mark the concentration of the LED lamp as β, the width of the mine cage is *l*, and the height of the mine cage as *H*. Thereby the condition that the range of the LED light irradiating the bottom of the cage door needs to be met is
    (7) W=2Htanβ,W≥l

### 3.3. Safe Area Delineation

**Figure 8:**
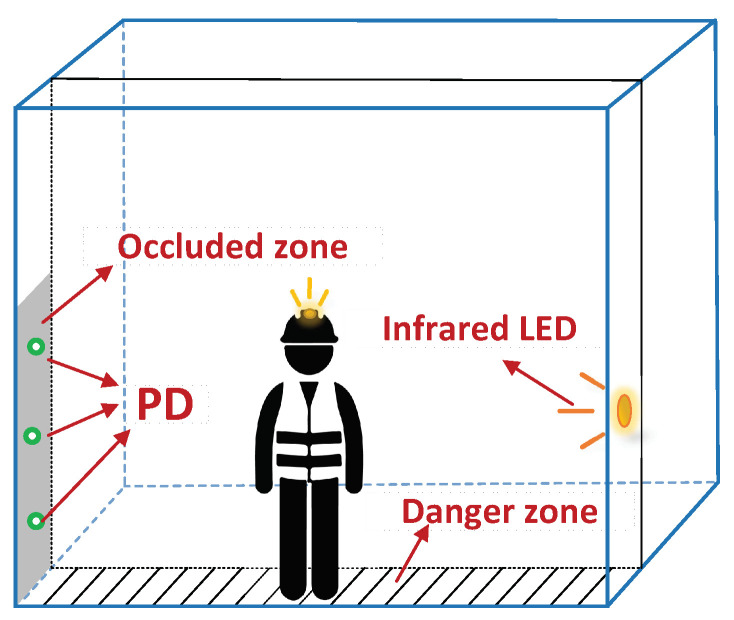
Monitoring whether personnel are in danger zone.

In addition to the dangerous situation where the limbs extend out of the mine cage, the hidden safety hazards including the falling of objects and the improper closure of the cage door also cannot be ignored. In order to minimize the probability of the aforementioned safety hazards, we define the safe area and restrict the movable range of personnel in the cage without affecting the normal riding of the cage (i.e., sacrificing cage space to ensure the safety of personnel in the mine cage). Except for the cage door, the other three sides of the mine cage are completely closed, so only one dangerous area needs to be defined on the side of the mine cage door to ensure the safety of miners [8,9]. This problem can be regarded as a two-dimensional positioning problem, locating whether the miners are in the safe area of the mine cage. However, considering the actual environment of the mine cage, it is difficult for traditional positioning methods (e.g., angle of arrival (AOA) and received signal strength (RSS)) to exert their advantages. Therefore, we designed a simple and efficient safe area monitoring method to determine whether the people are in the safe area of the mine cage. As shown in Figure 8, we use some PDs and the modulated LED lamp deployed on the sidewall of the cage for safe area monitoring. In order to avoid the interference of the lights in the safe area to the personnel in the mine cage, here we choose the infrared LED lamp as the transmitter, which is invisible to the human eye [41,42]. The specific implementation steps are similar to Section 3.2, including two steps: We first collect the frequency power of the modulated infrared LED lamp perceived by the *m*-th PD when no one is in the danger zone (i.e., $P_{m}^{rf}$). Secondly, when the mine cage is in operation, it is assumed that the frequency power of the modulated infrared LED lamp sensed by the *m*-th PD is $P_{m}^{rf}\left(t\right)$. It should be noted that the frequency selection method of the infrared LED lamp is the same as the frequency selection method for limb extension monitoring (see Section 3.2), and the two frequencies cannot be the same. Thus similar to Equation (4), the frequency power change ratio of the *m*-th PD is:

(8) $$P_{m}^{rf-change}=\frac{P_{m}^{rf}-P_{m}^{rf}\left(t\right)}{P_{m}^{rf}}\times 100\%$$

Similarly, we think someone is in the dangerous area when any PD senses a change ratio of the frequency power greater than 20%. We do not need to deploy too many PDs due to the large range shielded by personnel. Moreover, not only the similarity between limb extension monitoring and danger zone monitoring methods, but also the unique PWM frequencies of LED lights provide convenience for sharing the hardware of limb extension monitoring to achieve danger zone monitoring.

## 4. Experimental Evaluation

### 4.1. Experimental Equipment and Environment

In this subsection, we introduce the experimental equipment and environment. The experimental equipment is divided into two parts by default: the transmitter and the receiver. The experimental environment was the model of the mine cage.

**Figure 9:**
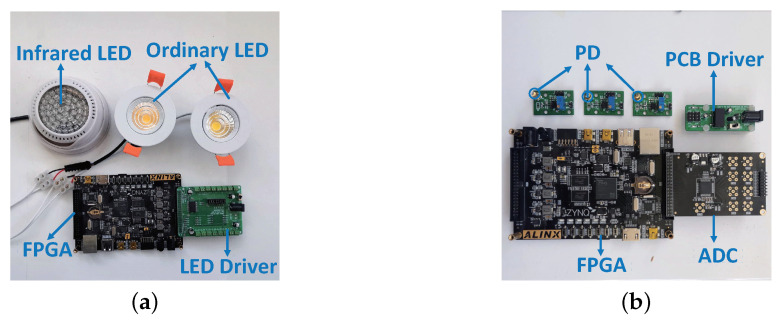
(**a**) Transmitter and (**b**) receiver.

**Transmitter** First of all, we chose commercial LEDs as the miner’s headlamps and the miner’s lamps. It had a wattage of 7 W and a spotlight angle of $30^{\circ }$. The lighting intensity and lighting range can fully meet the needs of the miners in general underground mines. Since the light emitted by the miner’s headlights was horizontal, we can make it emit a part of the light vertically upwards through a simple modification of the headlights to make the PDs perceive the light signal, e.g., by adding a small reflective surface under the headlamp. Secondly, we used a self-made LED driver board to supply power to these commercial LEDs. The output voltage of the driver board was 12 V, which can simultaneously control and supply power to eight of the above LEDs. Finally, we selected Xilinx ZYNQ-7020 FPGA as the modulator for the modulation of the PWM frequency and duty cycle according to our existing conditions. The actual equipment is shown in Figure 9a.

**Receiver** First of all, we chose the PD Honeywell SD5421-002 as the receiver. An FoV of $18^{\circ }$ and a rise time of 7 ns means a wider perception range and a higher frequency reception range (1/7 ns = 142.86 MHz). Next, we integrated it with a 10 KΩ adjustable resistor and LM358 amplifier in series to the printed circuit board (PCB) to avoid sensor saturation and enhance signal strength. Then we used the self-made PD driver to power it, which can simultaneously control and supply power to the four aforementioned circuit boards. Finally, we still chose Xilinx ZYNQ-7020 FPGA and the AN706 digital-to-analog converter (ADC) for data sampling and processing. Among them, the sampling frequency of AN706 is up to 200 KHz, which can simultaneously sample eight channels. The actual equipment is shown in Figure 9b.

**Figure 10:**
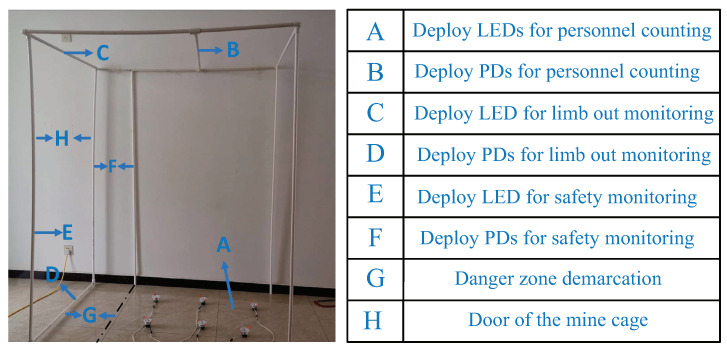
Mine cage model.

**Experimental environment** First of all, we built a 1.6 m × 1.2 m × 2 m mine cage model utilizing plastic pipes, and the largest number of people it can transport was six. Considering the dark mine environment, we placed it in a closed dark room. Secondly, when deploying PDs in the cage model, we selected four PDs for the limb extension monitoring, three PDs for the personnel counting, and three PDs for safe area monitoring according to the conditions we introduced before. Finally, the various parts of the cage model and their corresponding functions are given in Figure 10. It should be noted that except for the cage door, the surroundings of the cage were closed, although we have not reflected it in this mine cage model.

### 4.2. Personnel Counting

**Figure 11:**
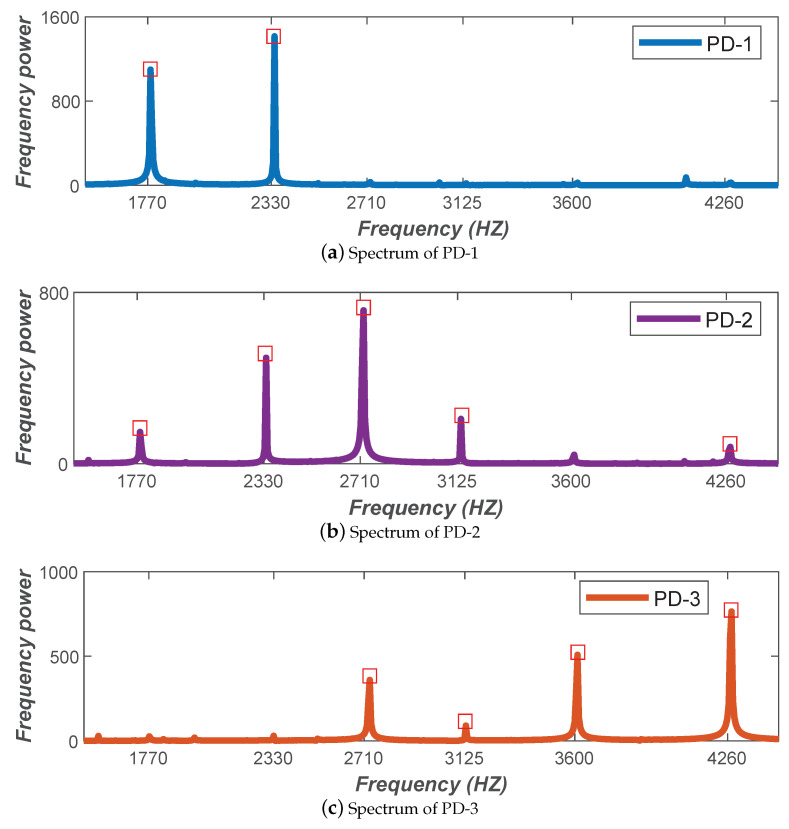
Personnel counting effect.

We placed six modulated LED lamps in the delineated safe area to represent the six miners according to the maximum number of people our mine cage model can carry, and the corresponding six visible light frequencies (Table 1) were selected to be allocated to all LED lamps according to the frequency selection method described in Section 3.1.1. Then, three PDs (denoted as PD-1, PD-2, and PD-3) were used on the top of the cage model to perceive the mixed light signals of the above six LED lamps. The experimental results are shown in Figure 11. It can be seen that each PD receives different series of frequencies within its perception range, and all our modulated visible light frequencies are included in the spectrum of the three PDs. Thus combined with multiple PDs, our algorithm can achieve accurate personnel counting.

### 4.3. Limb Extension Monitoring

**Figure 12:**
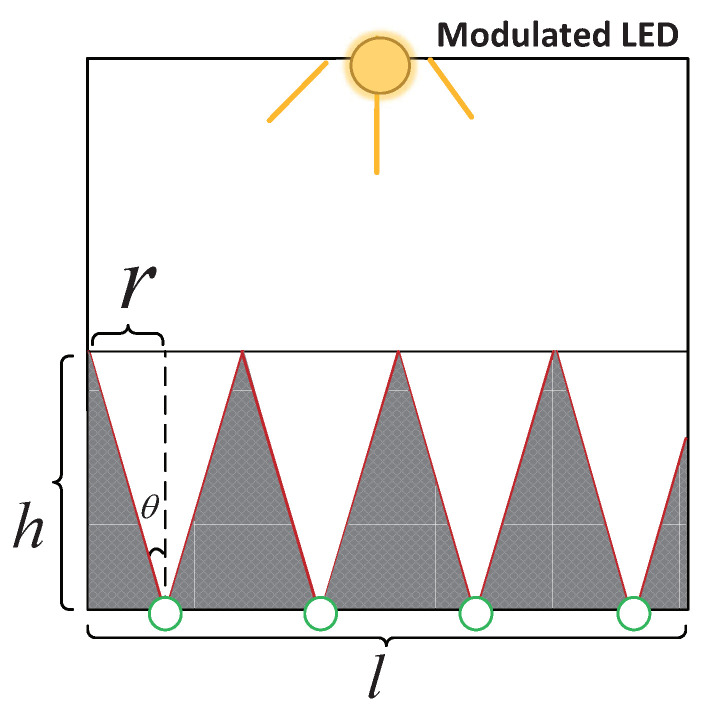
The precise location of the four PDs.

In this subsection, we verified the limb extension monitoring effect of our algorithm. We deployed four PDs as the receiver at the bottom of the simulated cage door and placed a modulation frequency LED lamp as the transmitter in the center of the cage door top. Firstly, the height of the simulated cage model we built was 2 m, and the concentration of the LED lamp used was $30^{\circ }$. We substituted the above parameters into Equation (7), and we then calculated that W>l, thereby the illumination of one LED lamp can fill the entire our simulated cage door bottom area. Secondly, we chose four PDs to be deployed at the bottom of the cage door. Since the width of the simulated cage was 1.2 m, and the FoV of the PD was $18^{\circ }$, then the area of this cage that we can fully monitor was 1–2 m according to Equation (5). The specific location of the four PDs placed on the mine cage door is depicted in Figure 12. In order to make full use of the sensing range of the PD, we first calculated the sensing radius (i.e., *r*) of the PD. As mentioned above, h=1 m, $\theta =9^{\circ }$, and r=htanθ, thereby we can calculate that *r* was approximately 0.16 m. Therefore, we only need to ensure that the interval of each PD was 0.32 m (i.e., 2*r*). Next, we tested the performance of the limb extension monitoring of our algorithm.

**Figure 13:**
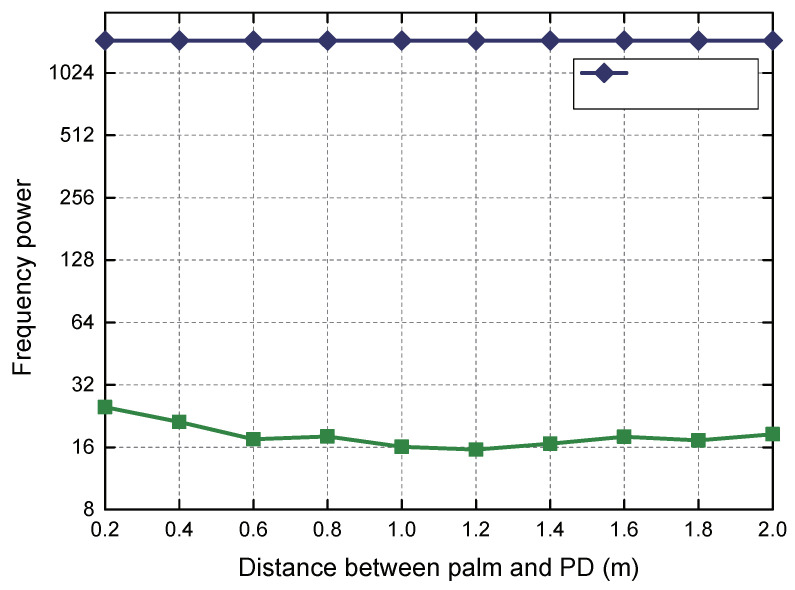
Different distances between palm and PD.

**Different distance between palm and PD** We first tested the effect of light blocked by limbs at different heights on the receiver. Since the PD can only perceive the change of light intensity within its FoV, we tested the relationship between the height of the light blocked by the palm and the ratio of power change when the PD and the LED lamp are vertically opposed (i.e., maximum height in the perception range). The experimental results are shown in Figure 13, and we can observe that when the palm blocks the direct path of the LED light irradiating the PD, the change ratio of its frequency power always fluctuates within a very small range and the average change ratio is 98.8%, regardless of the height of the palm and the PD.

**Figure 14:**
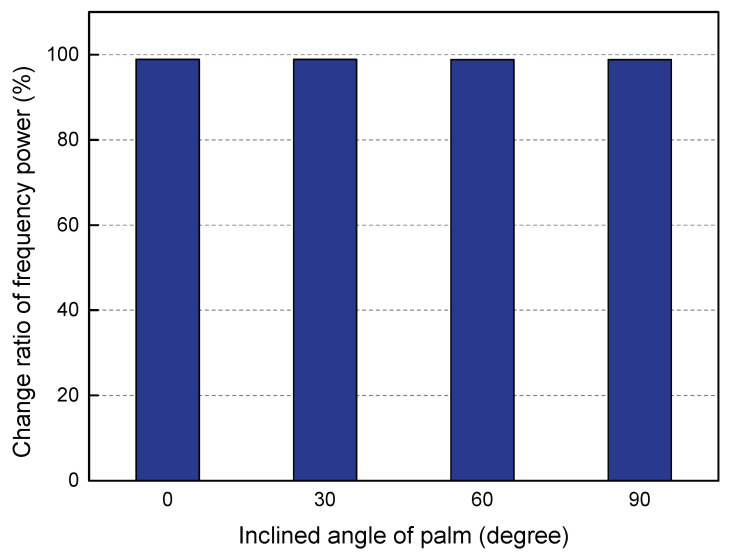
Different inclined angles of palm.

**Different poses of hands** Different hand postures directly affect the size of the area where the hand blocks light, which in turn affects the receiver’s perception ability. We tested the effect of different postures of palms on the monitoring effect of our algorithm. In order not to lose the generality, we divided the hand extension posture into three types, including horizontal (i.e., the inclination angle of the palm is $0^{\circ }$), inclined (inclined angle of the palm is $30^{\circ }$ and $60^{\circ }$), and vertical (inclined angle of the palm is $90^{\circ }$). Figure 14 plots the change ratio of the frequency power sensed by the PD at four different inclination angles of the palm (i.e., representing four different hand poses). We can intuitively observe that the frequency power change ratio in the above four cases is around 99%, which indicates that our algorithm can detect different gestures under the direct path of the PD and LED perfectly.

**Figure 15:**
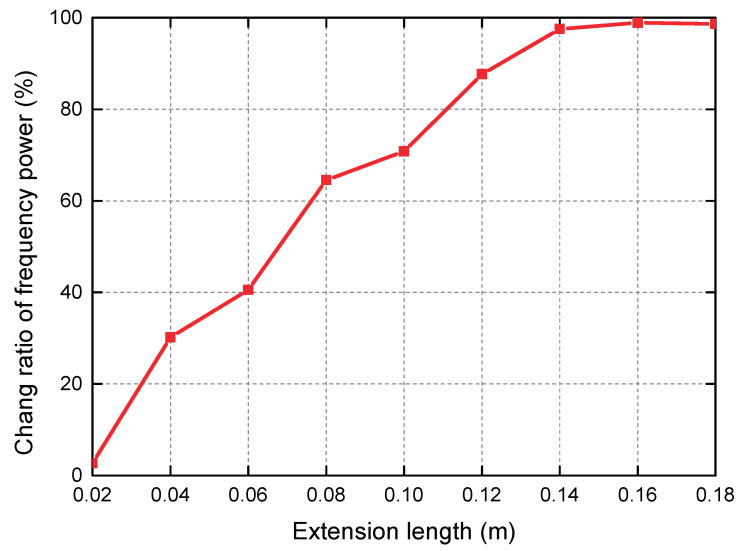
Different hand extension lengths.

**Extent of hand extension** Since the area of hands at different positions is different, i.e., the area of the finger part is relatively small, and the area of the palm part is relatively large, so now we test the change in frequency power when the hand is extended to different degrees. During the experiment, the length of the tester’s hand was 18 cm, of which the length of the finger was 8 cm and the length of the palm was 10 cm. After the hand extends out of the mine cage door, we collect data every 2 cm until the palm completely extends out of the mine cage door. Figure 15 plots the relationship between hand extension length and change ratio of frequency power. We can intuitively see that our algorithm is effective when the hand is extended by 0.04 m. The reason why 0.02 m does not meet the condition is because the ring finger is the longest, so the ring finger is also the first to extend.

**Figure 16:**
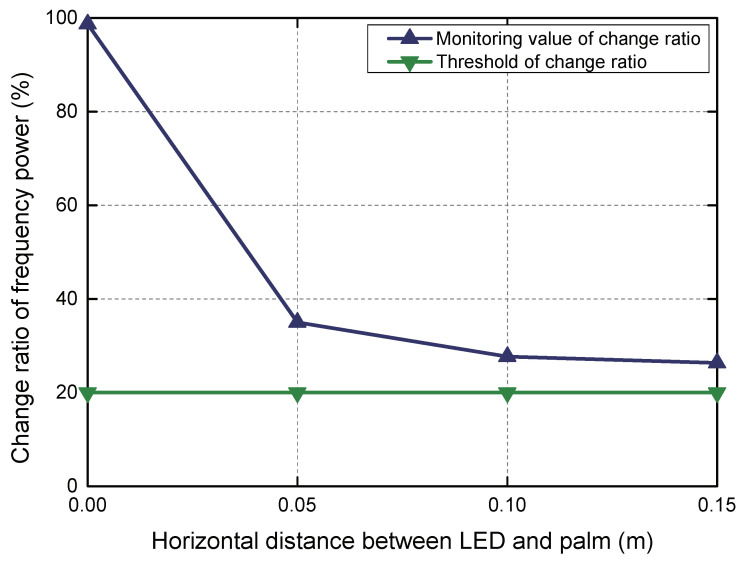
Different sensing areas of PD.

**Different sensing area of PD** We now discuss the performance of the PD to monitor the limb extension of the mine cage under the indirect path of the PD and the LED. We chose the vertical gesture with the smallest area to block light for this experiment, and its experimental results can fully represent that of the other gestures. Since the FoV of the PD was $18^{\circ }$ and the vertical height of the palm from the PD was 1 m, we calculated the radius of the horizontal perception range of the PD at the palm position to be 0.16 m (1×tan9=0.16). Therefore, we collected data every 0.04 m and plot the four sampling data in Figure 16. From Figure 16 we can see that the change ratio of frequency power decreases with the increase of the horizontal distance between the palm and the PD, and all meet the judgment condition of our algorithm to judge the limb extension monitoring (i.e., the change ratio of frequency power is greater than 20%). Thus we can claim that the PD has a good monitoring effect on different palm postures within its perception angle.

### 4.4. Safe Area Monitoring

**Figure 17:**
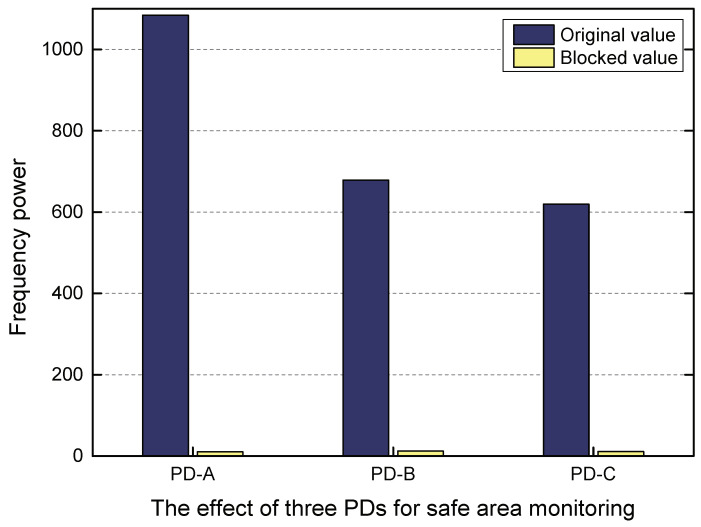
Safe area monitoring effect.

As mentioned earlier, three PDs and one infrared LED lamp were used for the safe area monitoring of our algorithm. We tested the changes in the frequency power of the infrared LED light monitored by three PDs when a person was in the dangerous area inside the cage. The experimental results are shown in Figure 17. We can see that when someone in the mine cage was in the designated dangerous area, the frequency power of the infrared LED light perceived by these three PDs changed greatly. It can be calculated that the change ratio of its frequency power was above 98% on average. Since the smaller danger zone we have defined and the larger space occupied by personnel, the infrared LED light can be completely blocked. Therefore, the probability that our algorithm monitors whether the person is in the delineated safe area can reach more than 99%.

## 5. Conclusions

Aiming to solve the challenging problem of the difficult safety monitoring of personnel overload and illegal limb extension in mine cages due to the harsh mine environment, we present a special mine cage safety monitoring algorithm utilizing visible light in this paper. This algorithm cleverly utilizes the existing lighting equipment of the mine cage and miners as the receiver to transmit optical beacon carrying the ID information of the miners through visible light frequency modulation, and then the cheap PDs were deployed in the mine cage to perceive the mixed optical signals. Finally, the methods of frequency matching and frequency power comparison were used to realize the efficient monitoring of personnel counting and limbs extending out of the mine cage, respectively. Moreover, We also designed a novel method of safe area monitoring to further reduce the probability of the limbs extending out of the mine cage. We built a simulated mine cage with a size of 1.6 m × 1.2 m × 2 m. Then, the combined transmitter (including Xilinx ZYNQ-7020 FPGA and commercial LED lights) and the combined receiver (including the PD Honeywell SD5421-002 and AN706 digital-to-analog converter (ADC)) were used to conduct the experiments to verify the effectiveness of our algorithm. The experimental results show that our algorithm achieves the accuracy of personnel overload and safe area monitoring of our algorithm can reach 99%, and the accuracy of limb extension monitoring is more than 96%. Finally, we believe that our algorithm will provide a new idea for the future of security monitoring in mine cages or other scenarios.

## Figures and Tables

**Table 1:** Six selected frequencies representing miners.

Miner	$m_{1}$	$m_{2}$	$m_{3}$	$m_{4}$	$m_{5}$	$m_{6}$
Frequency (Hz)	1770	2300	2710	3125	3600	4260
